# High‐throughput single‐molecule localization microscopy: Potential clinical applications

**DOI:** 10.1002/ctm2.1251

**Published:** 2023-04-24

**Authors:** Wei Shi, Shuang Fu, Yiming Li

**Affiliations:** ^1^ Department of Biomedical Engineering Southern University of Science and Technology Shenzhen China

## 1 GENERAL INTRODUCTION OF SMLM

**FIGURE 1:**
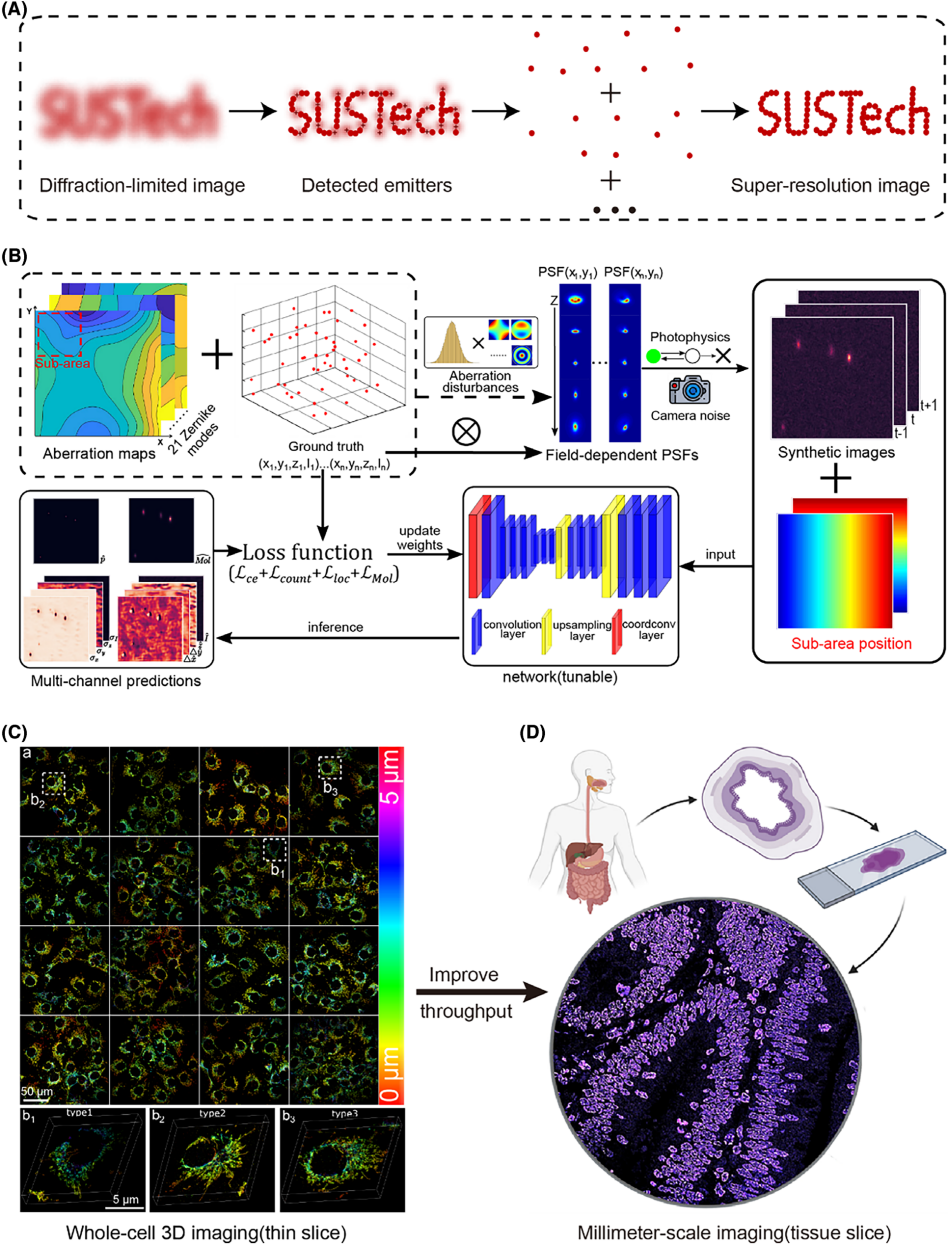
High‐throughput super‐resolution imaging. (A) The principle of SMLM, which reconstructs super‐resolution images by accurately localization of sparsely activated single‐molecule emitters. (B) Schematic of the FD‐DeepLoc, which encodes the spatial information in the neural network. (C) High‐throughput SMLM imaging example with quantitative analysis of whole cell 3D superresolved mitochondria. (D) Improving the imaging FOV to millimeter‐scale will enable super‐resolution imaging of tissue sections in clinical applications. Part D adapted with permission from REF.^7^

Single‐molecule localization microscopy (SMLM) has improved optical resolution to the range of several to tens of nanometres with high contrast, making it possible to noninvasively resolve nanoscale biological structures using fluorescence microscopy.^1^ In SMLM (Figure 1A), small subsets of fluorescent molecules are randomly activated to ‘blink’ due to the photon‐switching property of fluorophores. Due to the diffraction of light, each blinking event will form an image of point spread function (PSF), typically with a width of ∼200‐300 nm. After collecting several thousands of frames and computationally localizing all these sparsely activated molecules using a mathematical PSF model, a pointillistic super‐resolution image can be achieved, whose resolution is determined by both the localization accuracy and labelling density. Typically, ∼20 nm resolution can be achieved by SMLM, which is an order of magnitude improvement compared to the conventional optical microscope. Besides, through clever modulation of system PSF, SMLM can be extended to resolve three‐dimensional or even more information (e.g., colour, dipole orientation). Since it was first introduced in 2006, SMLM has revolutionized the field of biological imaging, allowing the visualization of subcellular organization with unprecedented detail. In the past ∼15 years, SMLM has been applied to investigate the organization, interaction, stoichiometry and dynamics of molecular machineries in cells and tissues, leading to tremendous new biological insights.^2^

## 2 THE BOTTLENECK OF HIGH‐THROUGHPUT SUPER‐RESOLUTION SCREENING FOR CLINICAL APPLICATIONS

The field of view (FOV) of super‐resolution microscopes is normally in the range of few tens of micrometres, which is much smaller than the size of clinical samples. Conventional high‐throughput super‐resolution imaging relies on an automated microscope to acquire small FOV one by one and stitch them to form a mosaic large FOV image by post‐processing. The imaging throughput is normally quite low, and some samples are not suitable for volumetric scanning. As SMLM is based on wild‐field detection, it has the potential to increase the imaging throughput just by using a camera with a larger detection array. This becomes feasible as the development of modern scientific‐grade complementary metal oxide semiconductor (sCMOS) camera technology, which offers ultra‐high number of pixels on a large chip in addition to high‐speed imaging and high‐sensitivity detection. Although sCMOS cameras with large detection array have promising high‐throughput screening potential for samples with large size, it was not fully employed due to optical aberrations. Modern super‐resolution microscopes normally are equipped with high numerical aperture (NA) objectives for better photon collection efficiency and attained resolution. For high NA objective lens, it is usually difficult to correct aberrations for areas far away from the central optical axis. As the FOV increases, field‐dependent aberrations become more pronounced and the PSF is spatially variant. Especially in the edge regions of the FOV far from the central optical axis, traditional SMLM reconstruction algorithms produce serious artifacts due to PSF model mismatch. Furthermore, the depth of field (DOF) of high NA objective is also very shallow, making it difficult to image samples with large volume. Therefore, 3D large FOV super‐resolution imaging of large‐scale clinical sample to utilize the full sCMOS camera chip was still very challenging.

## 3 FD‐DEEPLOC OVERVIEWS

In recent years, deep learning algorithms have been widely used in scientific research. With excellent feature perception and end‐to‐end image modelling capabilities, deep learning has demonstrated performance far exceeding traditional algorithms in SMLM. However, the traditional convolutional neural network uses the same convolution kernel for different regions in the image, which is not sensitive to the spatial position. As a result, it is not applicable to analyse the field‐dependent aberrations for large FOV imaging. To overcome this limitation, we proposed FD‐DeepLoc, a deep learning based single‐molecule localization framework, combining multi‐channel fitting^3^ and large DOF PSF engineering,^4^ for fast and precise localization of spatially variant single‐molecule point emitters.^5^ We introduced two position‐related channels in FD‐DeepLoc. When the convolution kernel convolutes single‐molecule images, it also convolves these two position channels, thereby encoding position‐related information into the neural network (Figure 1B). FD‐DeepLoc surmounts the insensitivity of traditional convolutional neural network to spatial position and accurately localize spatially variant single‐molecule data, realising high‐throughput 3D large FOV super‐resolution imaging with high fidelity. Through theoretical simulation and real biological samples, the performance of FD‐DeepLoc was much better compared to the previous algorithms. The root mean square error (RMSE) of 3D single‐molecule localization has been improved by nearly two times compared to the state‐of‐the‐art DECODE algorithm.^6^

Neuronal cells grow over hundreds of microns in culture. Conventional astigmatic 3D SMLM has very limited DOF (< 1.2 μm) and FOV (< 50 × 50 μm^2^), impeding the visualization of the 3D organization of different neurons in large scale. Due to the greatly improved imaging FOV afforded by FD‐DeepLoc, we could visualize the interaction of nerve cells across large FOV with high 3D resolution (30‐40 nm). The large FOV imaging also brings increased imaging throughput. Using FD‐DeepLoc, we could obtain hundreds of 3D super‐resolution images of whole cell mitochondria without manual selection in a short period of time. Through cluster analysis on the morphology of mitochondria (forks, length, sphericity, etc.), we classified the cells into three different types (Figure 1C). Type I cells contain more small round mitochondria and fewer branches; type II cells contain outspreading mitochondria and more complex networks; and type III cells contain a mixture of spherical and tubular mitochondria. These applications provided new ideas for super‐resolution cytomics.

## 4 OUTLOOK OF HIGH‐THROUGHPUT SUPER‐RESOLUTION IMAGING

As fluorescence microscopy with high contrast and super‐resolution has revolutionized structural cell biology studies, more and more attempt were made to apply super‐resolution microscopy in clinical studies,^7^, ^8^, ^9^, ^10^ such as detection of viral invasion receptors,^8^ analysis of pathological tissue for disease diagnosis,^9^ and quantification of antibodies on the surface of tumour cells during patient treatment.^10^ However, most of the current super‐resolution applications are limited to image single cells and thin pathological tissue. The throughput of SMLM is still insufficient for many clinical samples at millimeter‐scale (Figure 1D), such as exosomes, body fluid cytology, thick pathological tissue sections. Further increasing the throughput of SMLM to millimeter‐scale and efficiently generating high‐throughput super‐resolution images will make it a useful tool for clinical samples and open a new research window for investigating molecular mechanism of diseases using clinical samples directly from patients instead of cultured cells.

We believe that FD‐DeepLoc paved the way for extended high‐throughput super‐resolution imaging for clinical samples. High‐throughput super‐resolution imaging will enable researchers to visualise the structure and interaction of biological macromolecules, while analyzing the differences between cell populations in clinical tissue sections, providing a new perspective for the study of structural and cell biology. Thanks to its high molecular specificity and high‐resolution capability, it will provide a more reliable basis for clinical treatment and improve the level of disease diagnosis. Therefore, it will finally promote the widespread applications of SMLM technology in clinical research and diagnosis.

## CONFLICT OF INTEREST STATEMENT

The authors declare they have no conflicts of interest.
